# Suspected Hypersensitivity Reaction to Titanium Screws Following Kalish Osteotomy for Hallux Valgus

**DOI:** 10.7759/cureus.104709

**Published:** 2026-03-05

**Authors:** Samy Ajarrag, Marie-Christine Torchon

**Affiliations:** 1 Podiatric Medicine, Université du Québec à Trois-Rivières, Trois-Rivières, CAN

**Keywords:** hypersensitivity reactions, metal implant, sterile inflammation, surgery, titanium allergy

## Abstract

Titanium implants are generally regarded as biocompatible and inert; however, hypersensitivity reactions, although uncommon, have been increasingly recognized in orthopedic surgery. We report a case of chronic inflammatory reaction following a Kalish osteotomy for hallux valgus, ultimately attributed to titanium hypersensitivity. A 58-year-old woman underwent fixation of the first metatarsal with two titanium screws. The postoperative course was complicated by wound dehiscence, delayed healing, recurrent granulomatous tissue, and persistent swelling, despite antibiotic therapy. Over the months, two additional surgical procedures were performed, including the removal of a floating bone fragment, followed by biopsy and complete hardware removal. Histopathologic analysis demonstrated reactive bone changes without signs of inflammation or infection, ruling out osteomyelitis. After the removal of the titanium screws, symptoms gradually resolved. At four-year follow-up, the patient remained pain-free with stable bone alignment. This case emphasizes the importance of recognizing titanium hypersensitivity as a potential cause of chronic sterile wound dehiscence and inflammation following orthopedic implant surgery.

## Introduction

Titanium and its alloys are extensively used in orthopedic implants because of their exceptional mechanical strength, corrosion resistance, and high biocompatibility [1,2]. These properties have made titanium the preferred material for joint, spinal, and foot fixation devices, as it is generally considered biologically inert and unlikely to induce adverse immune reactions. Consequently, hypersensitivity to titanium has long been viewed as extremely uncommon [1,2]. Nonetheless, sporadic reports of unexplained postoperative inflammation and wound complications associated with titanium implants have begun to challenge this assumption [3-6]. Because routine laboratory and histopathological findings are often nonspecific, establishing a definitive diagnosis remains difficult and requires careful clinical, microbiological, and radiologic correlation [3,5-7]. The present report describes an unusual case of persistent sterile inflammation and wound dehiscence following fixation with titanium screws, illustrating how a seemingly inert biomaterial can trigger an unexpected and challenging postoperative course.

## Case presentation

**Figure 1:**
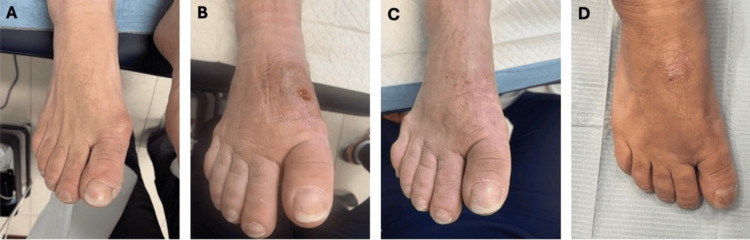
Clinical photographs of the patient’s right foot. A: preoperative appearance; B: wound healing complications with erythema and edema ten weeks after revision surgery; C: wound appearance at three-week follow-up after the third surgical intervention; D: four-year follow-up showing healed soft tissues at the surgical site.

A 58-year-old woman with a medical history of anxiety and mild eczema presented with progressive pain and deformity of the right hallux (Figure 1A). Clinical and radiographic evaluation confirmed stage III hallux valgus on the right, for which surgical correction was considered appropriate.

**Figure 2:**
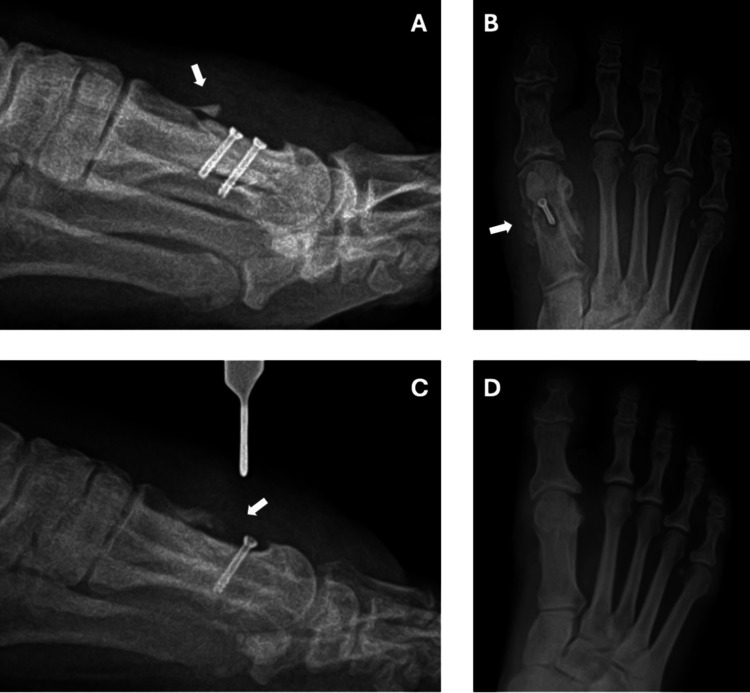
Radiographs of the patient’s right foot. A: four-week postoperative radiograph showing a small osseous fragment adjacent to the osteotomy site (arrow); B, C: radiographs obtained after revision surgery demonstrating persistent radiographic abnormalities, including focal bone erosion adjacent to the screw tract (arrows); D: four-year follow-up radiograph demonstrating complete osseous consolidation and resolution of prior abnormalities.

Surgical correction consisted of a distal Kalish osteotomy of the right first metatarsal performed under local anesthesia with ankle block. Fixation was achieved using two titanium alloy screws composed of Ti-6Al-4V. At two weeks postoperatively, wound dehiscence accompanied by localized erythema was observed, and a seven-day course of oral cefadroxil was initiated. Despite treatment, pain progressively worsened over the following weeks. At approximately four weeks postoperatively, persistent pain and erythema were noted. Sutures were removed, and radiographs demonstrated a small osseous fragment adjacent to the osteotomy site (Figure 2A), raising concern for an abnormal bone reaction. Empiric doxycycline therapy was subsequently initiated for presumed cellulitis.

Six weeks postoperatively, given the persistence of symptoms, revision surgery was performed. The procedure consisted of excision of hypergranulation tissue, removal of a small bone fragment, and extraction of the loosened screw. After the operation, erythema and edema remained, but there was no evidence of purulent drainage. At two weeks following the revision procedure, localized inflammation remained present. Laboratory investigations returned within normal limits, reducing suspicion for systemic infection.

At ten weeks following the revision surgery, due to persistent wound (Figure 1B) and radiographic abnormalities (Figures 2B-2C), a third surgical intervention was performed. This procedure involved the removal of the remaining titanium screw, excision of a pyogenic granuloma, bone biopsy, and culture sampling. Intraoperatively, yellowish discoloration of the surrounding tissues and focal bone erosion were observed. Histopathologic examination excluded osteomyelitis and malignancy, demonstrating no significant inflammation. Microbiological cultures yielded growth of *Staphylococcus* species; however, this was interpreted as probable contamination in the absence of histologic evidence of deep infection. The isolated organism was reported as sensitive to tetracyclines and cephalosporins on the antibiogram, both of which the patient had previously received empirically, and prior antibiotic exposure may have influenced microbiologic findings. Following complete hardware removal, local inflammatory changes gradually resolved (Figure 1C).

At long-term follow-up, the patient remained pain-free, with only mild residual edema and complete radiographic consolidation of the osteotomy (Figure 2D), accompanied by healthy overlying skin (Figure 1D).

## Discussion

Titanium is widely regarded as a biocompatible and inert material, which explains its extensive use in orthopedic implant surgery [1,2]. Nevertheless, cases of implant-related hypersensitivity have been increasingly reported in the literature [3,5,6]. Clinically, these reactions may closely mimic postoperative infection, presenting with erythema, edema, wound dehiscence, chronic sterile drainage, and persistent pain unresponsive to antibiotic therapy. Because hypersensitivity and postoperative infection share overlapping clinical and radiographic features, distinguishing between these entities remains particularly challenging [3,4,7].

The apparent inertness of titanium is attributed to the formation of a stable oxide layer [1,2]; however, several theories have been proposed to explain implant-related hypersensitivity reactions. Some authors suggest that the clinical syndrome observed around titanium implants may not represent a classic type IV allergic reaction, given the inert nature of titanium itself, but rather a distinct immunologic response potentially related to trace metals or contaminants present within titanium alloys [3,8]. Other hypotheses propose that mechanical stress, micromotion, and corrosion may promote the release of titanium ions or nanoparticles, which can bind to endogenous proteins and act as haptens, thereby triggering a delayed T-cell-mediated immune response [9]. In addition, titanium wear products have been shown to induce the release of pro-inflammatory cytokines, exert cytotoxic effects, and promote local bone resorption [10,11]. These mechanisms may result in a chronic peri-implant inflammatory reaction that persists despite the absence of overt infection or mechanical failure.

In the present case, sterile cultures, absence of purulence, normal laboratory findings, and non-specific histopathologic features were inconsistent with an infectious process. Histologic examination demonstrated reactive osteochondral changes without inflammatory infiltrate, foreign material reaction, or evidence of osteomyelitis. Similar histopathologic findings have been reported in cases of suspected titanium hypersensitivity, in which bone specimens often show reactive changes without signs of infection or malignancy [5,6]. Beyond wound-related complications, several studies have reported persistent postoperative pain and chronic localized inflammation related to retained implants [12,13]. In some reports, metal hypersensitivity has been suggested as a possible contributing factor when no other cause could be identified [3,7,14]. Several reports describe symptom improvement following implant removal once infection and mechanical failure have been excluded [3,5,7,15]. In this case, the patient’s clinical improvement after complete hardware removal is consistent with these observations.

The prevalence of titanium hypersensitivity remains poorly defined, largely due to inconsistent literature, as well as the absence of standardized diagnostic criteria and validated testing methods [3,4,7,16]. Although several diagnostic algorithms incorporating tools such as patch testing and the Memory Lymphocyte Immunostimulation Assay (MELISA) have been proposed [7,16], their limited sensitivity, specificity, and reproducibility restrict their routine clinical applicability [3,4,17-20]. As a result, orthopedic surgery still lacks standardized diagnostic or management guidelines. An expert consensus by Thomas et al. underscores the absence of standardized diagnostic criteria while encouraging clinicians to consider metal hypersensitivity in cases of delayed or unexplained postoperative complications [4]. Outside orthopedics, guidelines from the dental community based on systematic review and expert consensus have similarly concluded that neither epicutaneous testing nor lymphocyte transformation testing should be used to diagnose titanium hypersensitivity, favoring a primarily clinical diagnosis [20]. As a result, clinical decision-making continues to rely largely on individual clinician judgment and evidence derived from case reports and narrative reviews.

## Conclusions

Titanium hypersensitivity, although rare, represents an important differential diagnosis in chronic postoperative inflammation following orthopedic or podiatric implant surgery. The absence of infection and the presence of sterile granulomatous tissue or delayed healing should alert clinicians to this possibility. Early identification and hardware removal can result in resolution and prevent unnecessary antibiotic exposure. Greater awareness and standardized clinical approaches are needed to improve the detection and management of suspected metal hypersensitivity in surgical practice.
